# Development and function of the fetal adrenal

**DOI:** 10.1007/s11154-022-09756-3

**Published:** 2022-10-18

**Authors:** Emanuele Pignatti, Therina du Toit, Christa E. Flück

**Affiliations:** 1 Department of Pediatrics, Division of Endocrinology, Diabetology and Metabolism, University Hospital Inselspital, University of Bern, 3010 Bern, Switzerland; 2 Department for BioMedical Research, University Hospital Inselspital, University of Bern, 3010 Bern, Switzerland

**Keywords:** Adrenal development, NR5A1, Fetal-placental unit, Steroidogenesis, C11-oxy androgens, Cortisol, Sex differentiation

## Abstract

The adrenal cortex undergoes multiple structural and functional rearrangements to satisfy the systemic needs for steroids during fetal life, postnatal development, and adulthood. A fully functional adrenal cortex relies on the proper subdivision in regions or ‘zones’ with distinct but interconnected functions, which evolve from the early embryonic stages to adulthood, and rely on a fine-tuned gene network. In particular, the steroidogenic activity of the fetal adrenal is instrumental in maintaining normal fetal development and growth. Here, we review and discuss the most recent advances in our understanding of embryonic and fetal adrenal development, including the known causes for adrenal dys-/agenesis, and the steroidogenic pathways that link the fetal adrenal with the hormone system of the mother through the fetal-placental unit. Finally, we discuss what we think are the major open questions in the field, including, among others, the impact of osteocalcin, thyroid hormone, and other hormone systems on adrenal development and function, and the reliability of rodents as models of adrenal pathophysiology.

## Introduction

The adrenals are maybe the most fascinating organs of the human body with the structure and function of the fetal adrenals being significantly different compared to the postnatal and adult organs [1]. While the fetal adrenals are quite large organs that appear early post conception to produce predominantly adrenal androgen precursors, the adult adrenals are relatively small with a cortex that is formed of two layers after birth, producing mineralocorticoids (MC) in the zona glomerulosa (zG) and glucocorticoids (GC) in the zona fasciculata (zF). A third layer, the zona reticularis (zR), is gradually formed in the first years of life and only becomes functionally active in the production of adrenal androgen precursors at adrenarche around the age of 8 years. This innermost layer of the adrenal cortex also decreases its hormone production with senescence in the event of adrenopause, while GC production is quite constant during the entire lifetime [2, 3].

Although fetal adrenals are relatively large organs that function in a concerted network with the placenta and thus the mother, fetuses without adrenals develop, for all intents and purposes, normally until birth unless the underlying cause of adrenal aplasia is associated with anomalies of other organ systems. By contrast, adrenal insufficiency, in essence MC and GC deficiency, will cause potentially life-threatening water and electrolyte disturbances at any age postnatally [4]. In newborns which lack proper adrenal function, these crises occur typically in the second week of life, after the newborn is running out of maternal derived hormone reserves and is supposed to produce steroid hormones independently. During pregnancy the fetal adrenals form a complex functional steroid unit with the placenta and the mother’s steroids in circulation, the so-called fetal-placental-maternal unit, which – if disturbed – may harm both the fetus and the mother, predominantly through androgen excess [4, 5].

Although we have learned a great deal, concerning human adrenal development and function, from human (genetic) disorders in recent years, we still do not understand the intricate details of fetal adrenal development and function, its transition at birth and the cortex zonation, maintenance and renewal throughout life [6]. Studies of the adrenals have always been hampered by the fact that its structure and function are specie specific. Only higher primates share a similar structure and function of the adrenal cortex with humans, while rodents show remarkable differences in both structure and function that need to be considered when they are investigated as models. Nevertheless, much of the current knowledge of fetal adrenal development derives from mice models [7]. Only more recently, spatial and temporal gene expression profiling using single-cell sequencing on human fetal material has provided additional insight into developmental pathways [8, 9], and reprogramming of human induced pluripotent stem cells towards adrenal cell lines or construction of adrenal organoids are currently hot topics in the field [10, 11]. In addition, steroid profiling of available biomaterials using newer, comprehensive steroid analytical methods, for example sophisticated combined chromatography, and mass spectrometry approaches, have enhanced our insight into the evolution of fetal adrenal steroid biosynthesis and metabolism [12, 13].

In this article we summarize the current knowledge of the development and function of the human fetal adrenals from conception to the transition to the adult postnatal organ with birth. We also give a short overview on related adrenal disorders.

## Structural development of the fetal adrenal and its transition to the adult organ

**Fig. 1:**
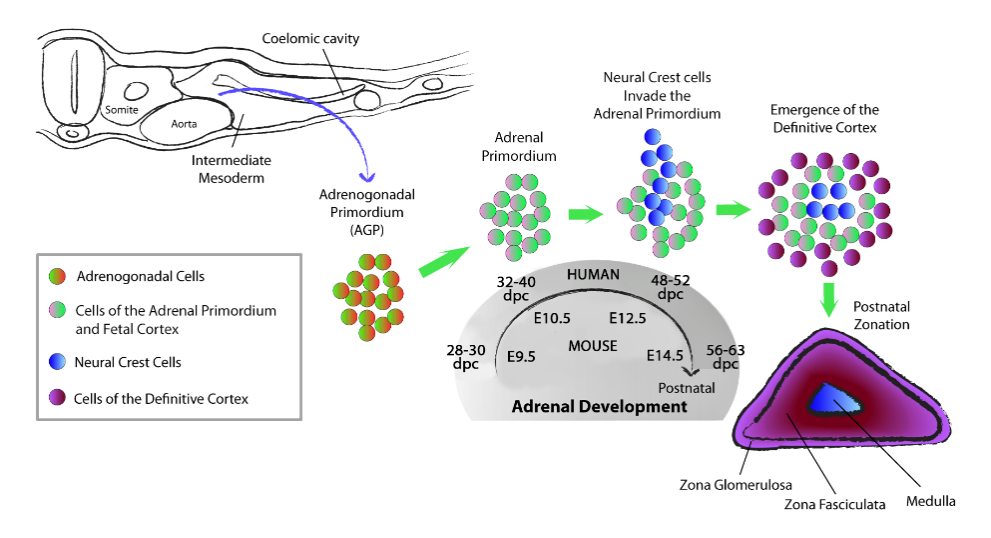
Schematic illustration of the cellular rearrangements during adrenal development in mice and humans. The adrenogonadal primordium (AGP) originates from cells localized between the coelomic epithelium and the dorsal aorta (blue arrow) at around 28–30 dpc (E9.5 in the mouse). At 32–40 dpc (E10.5 in the mouse), the adrenal primordium separates from the AGP. Between 48 and 52 dpc (E12.5), neural crest cells, precursors of the chromaffin cells of the medulla, invade the AGP. From 56 dpc (E14.5) onward, the fetal cortical cells are replaced by the definitive cortex, which gives rise to the adult zG and zF around the time of birth

The steroidogenic function of the adult human adrenal cortex is compartmentalized into three concentric regions, or ‘zones’, each responsible for the biosynthesis of distinct steroids [6, 14]. This structure allows for the discrete regulation of each steroid type and of the associated physiological function. While the adrenocortical structure is highly dynamic in response to systemic needs and alters with ageing, local signaling pathways persist throughout the adrenal lifespan to maintain the tripartite subdivision and a relatively standard adrenal size. Within the following paragraphs, we will show how this tissue architecture is the result of multiple cell rearrangements and migration events that begin in the fetus around 30 days-post-conception (30 dpc) in humans, and around embryonic day 9 (E 9.0) in the mouse (Table 1**and** Fig. 1), the animal model in which adrenal development has been mostly explored and on which most textbooks rely for knowledge on the subject. The mechanisms of early adrenal development in human have only recently begun to unfold [8], and the primary focus is on the early stages of adrenal specification and determination due to the restricted availability of human material at later time points.

**Table 1:** Key steps of embryonic adrenal development in the mouse and corresponding human developmental stages. Details about expression and transcriptional regulation of *NR5A1*/SF1 are also reported

Human embryonic stage	Mouse embryonic stage	Developmental events	SF1 expression
30 dpc	E9.0	The Adrenogonadal Primordium (AGP) is visible	- SF1 is not expressed
32–40 dpc	E10.5	The Adrenal Primordium (AP) separates by migrating in a dorsomedial position	- SF1 expression is initiated by the Hox-PBX1-Prep1 transcriptional complex on the FAdE promoter and then sustained by SF1 itself in a positive autoregulatory loop
48–52 dpc	E12.5	- Neural crest cells migrate into the adrenal anlage - The adrenal primordium is encapsulated - Two morphologically distinct regions are observed: the Definitive Zone (DZ) and the Fetal Zone (FZ)	- SF1 expression is sustained by SF1 itself in a positive autoregulatory loop
56 dpc = 8 wpc	E14.5		- FAdE promoter is inactive

Footnote: dpc - days post conception; wpc – weeks post conception

In the mouse, the adrenal glands derive from bilateral and symmetric streaks of intermediate mesoderm cells included between the dorsal aorta and the coelomic epithelium, clearly visible at E9.0 [15] (Table 1). These cell domains are referred to as the adrenogonadal primordium (AGP) and express the transcription factors GATA binding protein 4 (GATA4), Wilms’ tumor 1 (WT1) and the Cbp/p300-interacting transactivator 2 (CITED2) [16, 17]. The importance of WT1 and CITED2 for adrenal development is underscored by the consequences of their constitutive inactivation, which results in adrenal dys-/agenesis combined with other defects in organogenesis [18–20]. The impact of GATA4 at this developmental stage has not been investigated, but its inactivation at later stages has minor impact on adrenal development, possibly because of functional redundancy with the co-expressed GATA binding protein 6 factor [21].

At E10.5 in the mouse the AGP splits and gives rise to two independent population of cells corresponding to the gonadal (dorsolateral position) and adrenocortical (dorsomedial position) lineages, named the gonadal (GP) and the adrenal primordia (AP), respectively (Fig. 1). While the expression of CITED2 and GATA4 in the AP persists, WT1 is suppressed for adrenal differentiation to proceed [16, 17]. Indeed, stabilization of WT1 precludes adrenal differentiation through the upregulation of the direct targets transcription factor 21 (*Tcf21*) and glioma-associated oncogene 1 (*Gli1*), which are established markers of progenitor cells in both the developing and adult adrenal [16]. The importance of WT1 within the narrow timeframe of early adrenal development is associated with its ability to stimulate the expression of the nuclear receptor subfamily 5 group A member 1 (*NR5A1*) gene, encoding the steroidogenic factor-1 (SF1) [19, 20, 22, 23].

While the formation and division of the AGP is well established and characterized in mice, the unique aspects of adrenal specification in humans have been dissected only recently [8]. Using immunofluorescence and single-cell transcriptomics on embryonic material, Cheng et al. found that the specification of adrenal and gonadal precursors in humans does not occur within a common progenitor anlage, but instead takes place at two distinct regions of the coelomic epithelium (anterior and posterior, respectively), and at different developmental stages (30 dpc and 33 dpc, respectively). Consistent with this notion, the regionalization of gonadal and adrenal precursors is underscored by the expression of a different homeobox (HOX) gene code. Besides, the adrenogenic epithelium displays a unique expression signature devoid of GATA4 and is characterized by low levels of CITED2, a further deviation from the mouse model. Nonetheless, as in the mouse, a strong SF1 expression characterizes the human adrenal precursors. Finally, around 33 dpc, the human adrenal and gonadal progenitors are visible as distinct primordia (AP and GP) following the migration of coelomic cells due to a mechanism of epithelial-to-mesenchymal transition [8].

SF1 is a member of the nuclear receptor superfamily and drives the differentiation of multiple organs in mice and humans, including the adrenals, gonads and spleen [24]. Opposite to WT1, CITED2 and GATA4, whose expression in steroidogenic cells are confined to embryonic stages, SF1 displays a robust expression in both embryonic and postnatal adrenals in both mice and humans. SF1 acts as master regulator of steroid biosynthesis by driving the transcription of a range of cytochrome P450 steroid hydroxylases involved in steroidogenesis [25]. During early adrenal development in mice, SF1 dosage is responsible for specifying the fate of AGP cells. Specifically, Val and colleagues showed that CITED2 potentiates the transcriptional activity WT1, which results in a higher SF1 expression in the AP with respect to the GP [17]. This and other studies in the mouse [26, 27] indicate that high SF1 levels are needed for proper adrenal development, while sex determination and the majority of gonadal development are not affected by SF1 haploinsufficiency, which indicates that adrenal development is more sensitive to SF1 dosage than gonadal development in the mouse. Altogether, these findings suggest that adrenal and gonadal specification in the mouse relies on a threshold of SF1 expression, whereas high SF1 levels are associated with the development of adrenocortical cells. The differential expression of SF1 in AP and GP is also present in humans despite the low expression of CITED2, suggesting that additional, yet-unknown mechanisms regulate the transcription of the human *NR5A1* gene. However, contrary to the mouse, it is unlikely that a dosage-dependent mechanism explains lineage specification in humans, where gonadal development and function is more commonly affected than adrenal function even in cases of SF1 haploinsufficiency [28–32]. Therefore, further investigation is needed to address the contribution of SF1 levels to human adrenogonadal specification and determination.

Due to its prominent role as a steroidogenic regulator, SF1 has been regularly used as a reprogramming factor for generating adrenal/steroidogenic cells from mesenchymal and induced pluripotent cells of different animal origins, including human [33]. These reprogramming efforts, ultimately intended as alternative regenerative medicine approaches to adrenal insufficiency, contributed to validate the role of SF1 as master regulator of steroidogenic differentiation and prompted new studies to interrogate the transcriptional regulation of *NR5A1*. In this direction, Zubair et al. identified a fetal adrenal enhancer (FAdE) in intron 4 of the murine *Nr5a1* gene, responsible for the initial production of SF1 in a WT1-independent manner under the control of a HOX-PBX1-PREP1 transcriptional complex, and later controlled by SF1 itself in a positive autoregulatory loop [34]. Finally, at around E14.5, SF1 SUMOylation and the recruitment of the SF1 inhibitor DAX1, encoded by the *Nr0b1* gene, contribute to terminating the *trans* activity of SF1 on the FAdE promoter, while SF1 expression persists [35]. The nature of SF1 transcriptional regulation at later developmental stages in the fetus and in the postnatal adrenal remains unknown. Besides, while the FAdE locus in mice shares a high degree of homology with the human sequence, the activity of the putative human promoter has not yet been tested [34].

At around 48–52 dpc in human (E12.5 in the mouse) a subset of neural crest cells invades the AP and gradually coalesces in the center of the developing adrenal glands. These cells eventually differentiate into catecholamine-producing chromaffin cells and form the adrenal medulla (Fig. 1) [36]. At this same stage, the adrenal capsule forms from mesenchymal cells surrounding the AP and mesenchymal-like cells of the fetal adrenal [37]. After encapsulation, the adrenal anlage is stratified into two morphologically different tissues: an outer region of highly packed basophilic cells that constitutes the ‘definitive zone’ or ‘definitive cortex’ (DZ) and makes up about 20% of the cortical tissue, and an inner, larger ‘fetal zone’ (FZ) containing cells with a high cytoplasmic-nuclear ratio. A third region, referred to as the ‘transitional zone’ (TZ), develops between the DZ and FZ after mid-gestation [38, 39]. Pseudotemporal ordering of cell transcriptomes from human embryonic adrenal cells suggests that the DZ originates directly from cells of the AP, whereas the FZ can both derive from the AP or from DZ cells [8]. Finally, the FZ is destined to disappear by apoptosis soon after birth, resulting in an about 20-time decrease in adrenal mass from the 4–5 g full-term adrenal, and a rapid fall in the production of androgen precursors, namely dehydroepiandrosterone (DHEA) and dehydroepiandrosterone-sulfate (DHEA-S) [40, 41]. On the contrary, the DZ and TZ give rise to the adult adrenal cortex [42–45]. There is ongoing debate of whether FZ involution depends on parturition or gestational age. Ultrasonography in premature neonates suggested that FZ involution occurs soon after parturition independent of the gestational age [46]. Instead, comparison of longitudinal hormonal profiles from term and pre-term babies indicates that fetal typical androgen production, as a proxy for zF activity, declines in accordance with postmenstrual age independent of the timepoint of birth [47]. The failure of the FZ to undergo apoptosis could be linked to the occurrence of early-onset pediatric adrenocortical carcinomas, although this possibility needs to be formally tested [48, 49]. The different steroidogenic profiles of DZ, TZ and FZ and their impact on gestation will be outlined in the following section.

Studies in the mouse show that adrenocortical cellularity both during development and adulthood is maintained by two distinct mechanisms: the inherent proliferation of adrenocortical cells and the recruitment of progenitor cells from the adrenal capsule. These progenitor cells migrate centripetally within the cortical tissue and differentiate into cells of the steroidogenic lineage. The first evidence for progenitor cells in the capsule came from lineage tracing experiments revealing that GLI1-positive mesenchymal capsular cells at E14.5 give rise to clusters of GLI1-negative SF1-positive steroidogenic cells at later developmental stages and in the adult mouse [37, 50]. Also WT1- and TCF21-expressing capsular cells were shown to be able to differentiate into steroidogenic cells, with the exception that the latter can only contribute to the fetal steroidogenic pool before the time of encapsulation [16, 37]. Of note, there is little overlap between GLI1- and WT1-positive cells, suggesting a different developmental origin of these cell types [16]. The functional redundancy among all these progenitor pools is still not clear, and further investigation is needed to determine their relative contribution to fetal development. In addition, the contribution of these cell population to adrenal renewal in humans remains unknown.

Adrenal development also relies on multiple paracrine and endocrine factors that support cell proliferation and differentiation, and prompt the maturation of adrenal subsidiary structures, including the vascular bed and nervous ganglia. For instance, both the adrenocorticotropin hormone (ACTH) and Angiopoietin 2 promote angiogenesis in the developing adrenal, while also contributing to cell proliferation and stimulation of the secretory function [51–54]. The adrenal vascular system is characterized by arteriolar capsular plexa and anastomoses that collect blood from the left renal artery or the inferior phrenic artery, and then extend into sinusoidal capillaries that drain at the level of the medulla into a single vein [55]. The adrenal nerve terminals, derived from the splanchnic nerves, project through the adrenal along the blood vessels, suggesting a tight interdependence of these two structures during development [56]. Experiments in sheep revealed that during fetal maturation the splanchnic innervation sustain cortisol secretion upon acute hypotensive and chronic hypoxemic stressors, indirectly suggesting a vital role for adrenal nerve terminals during gestation [57–59]. Besides hormonal and structural support, the developing adrenals also benefit from the activation of local paracrine signals. The signaling pathway that best recapitulates this concept is activated by the Wingless/Integrated (WNT) pathway ligands, whereby ablation or constitutive activation of the essential WNT transducer β-catenin results in adrenal dys-/aplasia [60, 61].

While the structural features of the fetal adrenal are largely in place by the 8th week of gestation, steroid function undergoes important changes throughout the duration of the gestation to support fetal development, as reviewed in the following section.

## Fetal adrenal function and its role in the fetal-placental steroid unit

**Fig. 2:**
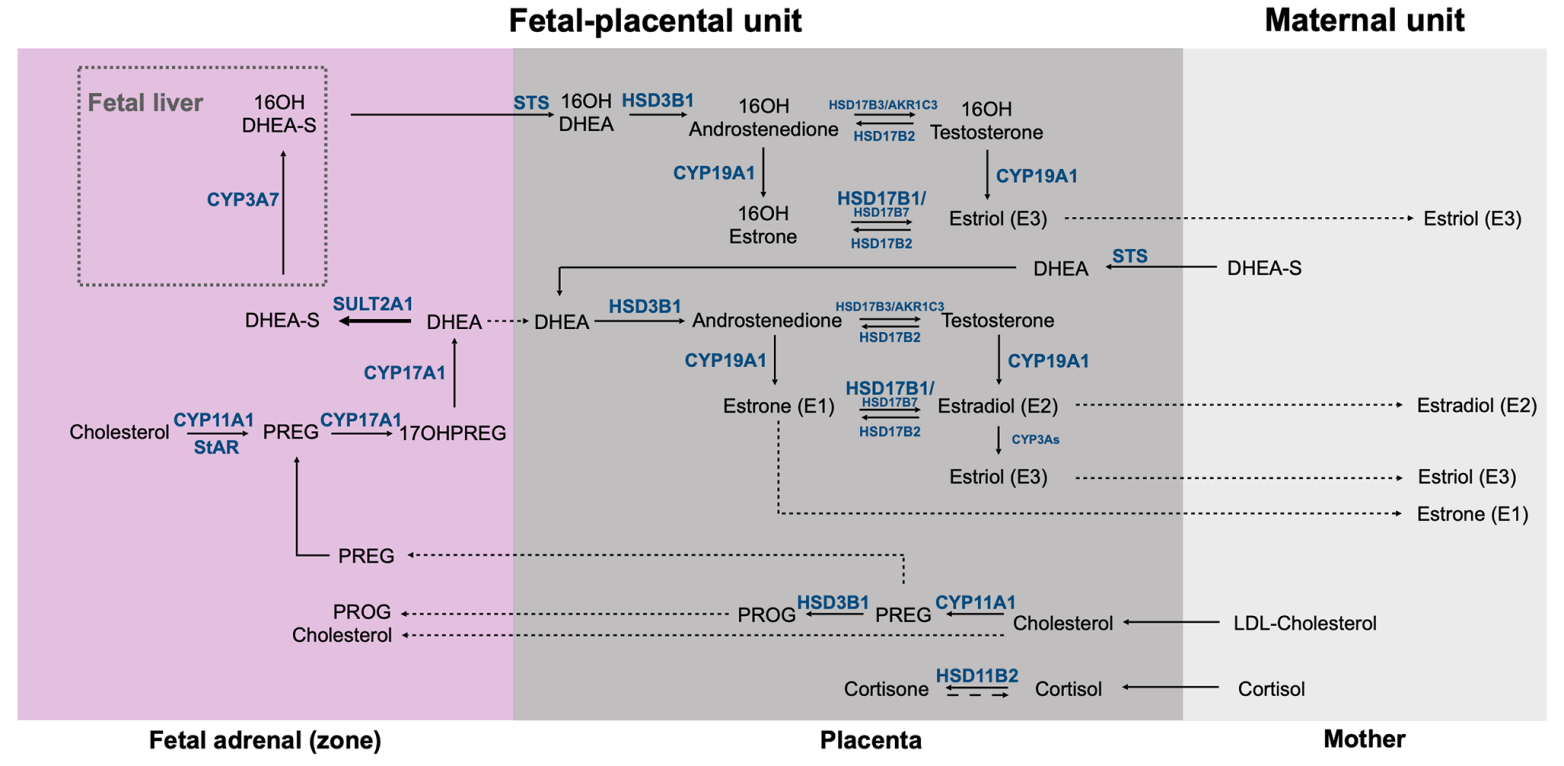
Steroid biosynthesis in the fetal adrenal, and steroid metabolism in the fetal liver and placenta, marking steroid metabolic pathways in the fetal-placental-maternal unit. PREG, pregnenolone; 17OHPREG, 17α-hydroxypregnenolone; PROG, progesterone; DHEA, dehydroepiandrosterone; DHEA-S, dehydroepiandrosterone-sulfate; CYP11A1, cytochrome P450 cholesterol side chain cleavage; StAR, steroidogenic acute regulatory protein; CYP17A1, cytochrome P450 17α-hydroxylase/17,20-lyase; SULT2A1, sulfotransferase; CYP3A7, cytochrome P450 family 3 subfamily A member 7; CYP19A1, cytochrome P450 aromatase; HSD17B1, 17β-hydroxysteroid dehydrogenase type 1; HSD17B2, 17β-hydroxysteroid dehydrogenase type 2; HSD17B7, 17β-hydroxysteroid dehydrogenase type 7; HSD17B3, 17β-hydroxysteroid dehydrogenase type 3; AKR1C3, 17β-hydroxysteroid dehydrogenase type 5; HSD3B1, 3β-hydroxysteroid dehydrogenase type 1; HSD11B2, 11β-hydroxysteroid dehydrogenase type 2; STS, sulfatase

Steroid hormones are vital in human development and are biosynthesized by the fetal adrenal cortex at critical stages during fetal development. While total steroid production in adult humans are, in principal, an accumulation of the contribution of the steroids from the adult adrenals and gonads and their metabolism, the fetal steroidogenesis is driven by the contribution of the fetal adrenals, the 46,XY gonad, the placenta and the shuttling of steroids from the mother through the placenta (Fig. 2).

**Fig. 3:**
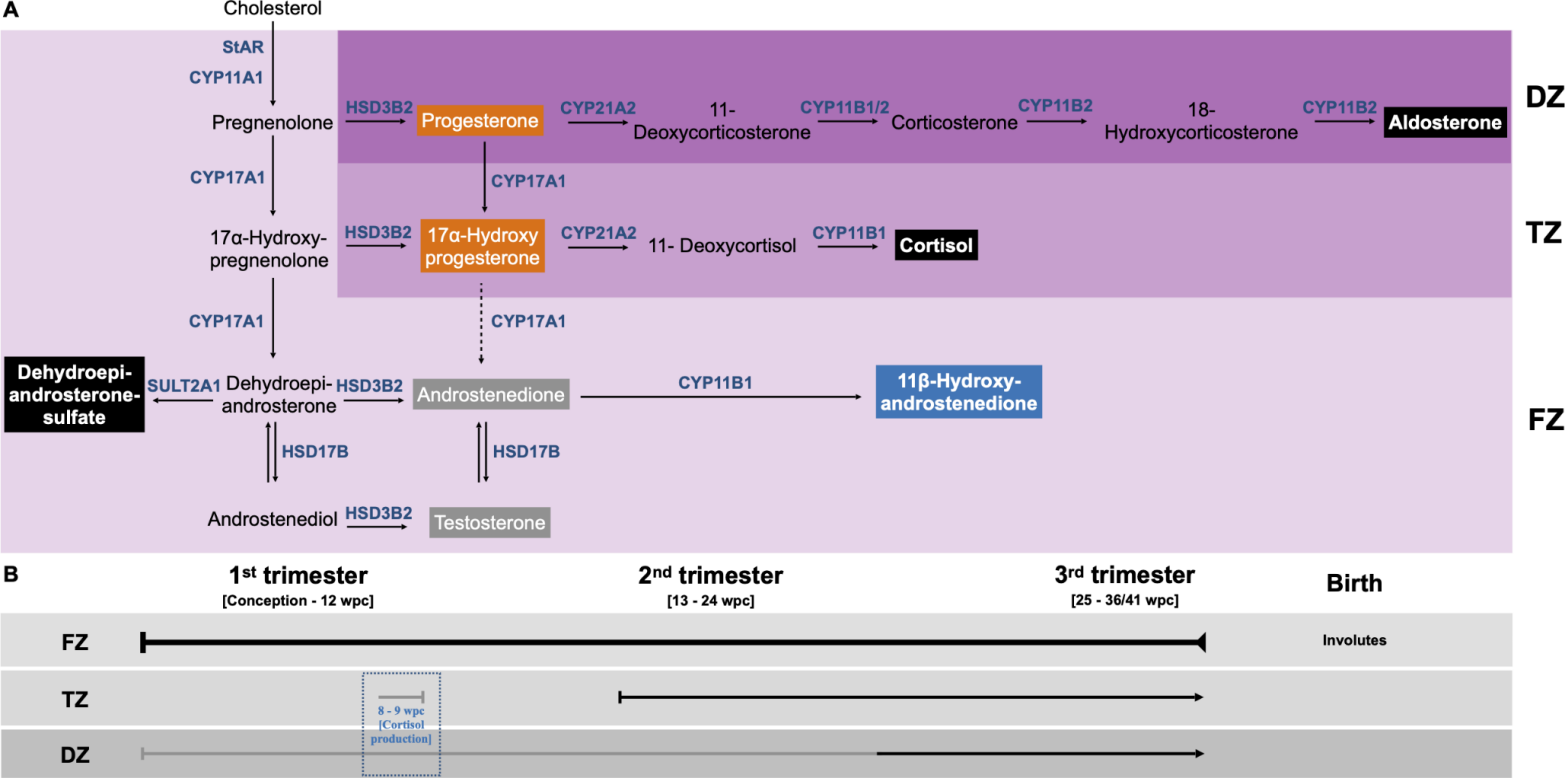
Steroid metabolic pathways in the fetal adrenal zones, **A**; with the developmental timeline of each zone during gestation, **B**. Steroidogenic acute regulatory protein (StAR), expressed in the fetal zone (FZ) (and transition zone [TZ]) from 6–8 wpc and in the definitive zone (DZ) only from 22–23 wpc; cytochrome P450 cholesterol side chain cleavage (CYP11A1), expressed in the FZ from 8–9 wpc and onwards, and in the TZ from 14–22 weeks, while the expression in the DZ appears only from 17–19 and 23 wpc; cytochrome P450 17α-hydroxylase/17,20-lyase (CYP17A1), expressed in the FZ (already from 8–9 wpc) and TZ from 14–24 wpc; 3β-hydroxysteroid dehydrogenase (3βHSD2) is expressed from 8–19 wpc in the DZ and from 24–41 wpc in the TZ; cytochrome P450 steroid 21-hydroxylase (CYP21A2) is sparsely expressed in the DZ between 13–24 wpc (only becoming prominent during late gestation) and detected in the FZ and TZ from 14 wpc (while detection in the FZ at 9–19 wpc has been shown); cytochrome P450 aldosterone synthase (CYP11B2), becomes prominent in the DZ during late gestation; and cytochrome P450 11β-hydroxylase (CYP11B1) is expressed in the fetal adrenal, in both the FZ and TZ by 13–24 wpc (limited weak staining in the FZ between 8–12 wpc has also been shown) [39, 43, 63, 64, 66, 90]. Steroids boxed in orange, steroid substrates for the backdoor pathway; steroids boxed in grey, steroid substrates for placental aromatase; steroid boxed in blue, steroid substrate for placental HSD11B2; steroids boxed in black, steroid end-products of each zone of the fetal adrenal

The main function of the FZ of the fetal adrenal is the production of DHEA-S from cholesterol (Fig. 3), which is transported to the placenta, desulfated to DHEA, and sequentially metabolised by placental 3β-hydroxysteroid dehydrogenase type 1 (3βHSD1), cytochrome P450 aromatase (CYP19A1) and 17β-hydroxysteroid dehydrogenase type 1 (17βHSD1), to androstenedione (A4), estrone (E1) and estradiol (E2), respectively (Fig. 2). E1 and E2 is then transported from the placenta into the maternal circulation, while maternal DHEA(S) also contribute to placental estrogen biosynthesis (about 40%) [62]. Fetal adrenal DHEA-S is importantly also metabolized by the fetal liver to 16α-hydroxy-DHEA-S by cytochrome P450 family 3 subfamily A member 7 (CYP3A7), which follows the same metabolic routine through the placenta as DHEA-S, and finally metabolises to estriol (E3), the estrogen marker of pregnancy (Fig. 2). Estrogens, together with GC and progesterone (PROG) metabolites, metabolized by the fetal-placental unit, maintain gestation and regulate fetal development [5, 45].

The earliest significant function of the fetal adrenals appears to be the timely production of cortisol between 8 and 10 weeks-post-conception (wpc) from placental PROG [63, 64], with *de novo* cortisol production also shown by 8–14 wpc [65, 66]. The placenta can convert maternal cholesterol to pregnenolone and PROG due to the expression of cytochrome P450 cholesterol side-chain cleavage (CYP11A1) and 3βHSD1. The subsequent steroidogenic enzymes necessary to produce cortisol, include cytochrome P450 17α-hydroxylase/17,20-lyase (CYP17A1), cytochrome P450 21-hydroxylase (CYP21A2) and cytochrome P450 11β-hydroxylase (CYP11B1), which have all been detected in fetal adrenal cells between 7.1 and 7.4 wpc at the interface between the DZ and FZ (Fig. 3), and in addition, positive immunostaining of 3βHSD type 2 (3βHSD2) and CYP11A1 was also detected. The staining for 3βHSD2 is abundant between 8 and 9 wpc, concurrent with the cortisol peak between 8 and 9 wpc, while it is not detected 5 weeks later (with the cortisol peak also declining after 8–9 wpc) [43, 67]. The authors suggest that the *de novo* production of cortisol during this crucial stage of fetal development negatively feed backs to the anterior pituitary corticotrophs and regulates the ACTH dependent androgen production in the female fetal adrenal, essentially safeguarding normal female sex differentiation [43, 68, 69]. In contrast to this safeguarding of female development, during the same time the fetal testis Leydig cells biosynthesize T from cholesterol and steroid-5α-reductase type 2 (SRD5A2), expressed in genital skin, converts T to dihydrotestosterone (DHT). T and more efficiently DHT, bind the androgen receptor (AR) in the testis which is expressed between 8 and 20 wpc, thus initiating the differentiation of male external genitalia [70]. Thus, research to date infer that the fetal adrenal has an important function in establishing sexual dimorphism in the first trimester of human development.

The most recent research – focusing on the function of the fetal adrenal – underscored androgen biosynthesis in the fetal-placental steroid milieu through the backdoor pathway and from adrenal 11β-hydroxyandrostenedione (11OHA4). Considering the latter, 11OHA4 is an endogenous hormone biosynthesized from A4 catalysed by adrenal CYP11B1 [71] (Fig. 3). Once 11OHA4 is produced, it is catalysed by 11β-hydroxysteroid dehydrogenase type 2 (11βHSD2) to 11keto-androstenedione (11KA4), and 11KA4 is a substrate for 17βHSDs, producing 11keto-testosterone (11KT), a potent androgen. Altogether these androgen precursors and androgens are termed C11-oxy androgens or 11-oxygenated androgens in literature published to date. Full-term placentas, sectioned from the fetal side, were analysed for androgen levels and 11KA4 was the most abundant measured androgen precursor. In addition, there were no differences in C11-oxy androgens levels between females and males, owning to their adrenal origin [72]. Due to the high 11βHSD2 activity in the placenta, 11KA4 and 11KT levels, as expected, were higher than 11OHA4 levels, and collectively 11KA4 and 11KT were measured at higher levels compared to A4 and T, in all likelihood due to these C11-oxy androgens not contributing to the estrogen pool [73, 74]. The presence of the C11-oxy androgens in the fetal-placental unit offers two possible explanations for their origin; either maternal adrenal 11OHA4 is converted to 11KA4 in the placenta, or 11OHA4 is biosynthesized in the fetal adrenal and then transported to the placenta where further metabolites are produced. As 11OHA4 has been measured in fetal adrenals at 16 wpc [63, 75] and in amniotic fluid [76], the latter is definitely possible. Confirming the results of the Yoshida et al. study [71], another research group also showed increased 11OHA4 and 11KA4 levels in maternal circulation from the first trimester compared to term, while 11KA4 was measured at higher levels compared to 11OHA4 and 11KT in neonatal cord blood at term [77]. The distinct role of these androgen precursors and androgens within the fetal-placental unit and their impact on normal fetal development has not yet been elucidated; however, their over-production has been suggested to cause fetal virilization. A clinical case study has reported the virilization of a female fetus due to a maternal androgen-producing adrenal tumour, and while 21-hydroxylase deficiency and cytochrome P450 oxidoreductase (POR) deficiency (PORD; presumably related to aromatase activity) were excluded as possible causes, levels of C11-oxy androgens were markedly elevated in the mother. In serum, 11KT levels were high, while T and A4 levels were within the normal references ranges and urinary C11-oxy androgen levels were also elevated. The authors concluded that the elevated production of androgenic 11KT crossed the placental barrier, causing the virilization; following removal of the tumour, indeed, C11-oxy androgen levels were markedly reduced [78]. Unfortunately, the authors did not measure steroid levels in the affected neonate, however, the authors do reference eight other children with 46,XX disorders/differences of sex differentiation (DSD), where the mothers also had androgen-producing adrenal tumors and only mildly elevated T levels, suggesting that alternative androgens may contribute to the virilization process [79–84].

Considering now the backdoor pathway, from the above mentioned study by Yoshida et al. [71], PROG, allopregnanolone and androsterone were also measured in placental tissue, with PROG being the precursor hormone for the backdoor pathway and both allopregnanolone and androsterone downstream metabolites of this pathway [85]. The backdoor pathway describes the production of DHT from PROG and 17α-hydroxyprogesterone (17OHPROG) (Fig. 3) through the combined enzymatic catalytic activities in the fetal liver, fetal adrenal, fetal peripheral/genital skin and the placenta [67, 86–90]. The concern with this pathway is the *in utero* virilization of female fetuses due the biosynthesis of an androgenic steroid which would compromise female sex differentiation. This is observed in PORD, where females virilize *in utero* due to decreased CYP17A1 activity and reduced placental CYP19A1 activity, while CYP21A2 activity is also attenuated, depending on the POR mutation [90–92], suggesting increased steroid precursors for conversion in the adrenal backdoor pathway are produced and turnover of androgens into estrogens are reduced [90, 93, 94]. Surprisingly, the backdoor pathway is commonly interrogated using the quantification of intermediate metabolites, especially androsterone, which has only a mild androgenic activity. Instead, the production of the more potent DHT is poorly covered in the literature [95]. The steroidogenic enzymes that catalyse the intermediate metabolism of PROG and 17OHPROG through the backdoor pathway on their way to DHT have been studied, but HSD17B6 (a retinol-like dehydrogenase, RoDH), which catalyses the key final step of androsterone conversion to DHT, lacks in-depth investigation possibly because it is only marginally expressed in the fetal-placental unit [90]. Remarkably, sex differences in the mRNA expression of key enzymes involved in the backdoor pathway have been shown in female and male fetal adrenal tissue between 6 and 10 wpc, suggesting that the backdoor pathway has a specific role to play in the female fetus, especially since the AR is expressed in female genital skin from the onset of sex differentiation [90]. Moreover, while the relevance of this pathway should still be explored in the normal fetus and the fetus with classic congenital adrenal hyperplasia, the testicular backdoor pathway and the contribution of the fetal adrenals to this pathway in terms of male fetus masculinization is a current topic of investigation in our and other research groups [87, 89].

MC and GC, produced within the DZ and TZ (Fig. 3), also play essential roles during middle-to-late gestation. Aldosterone is biosynthesized mainly during late gestation, and possibly earlier at lower levels [96], and its production becomes essential in post-natal life to prevent salt-wasting disorders. Instead, cortisol is detected during both the first (sex determination period) and the third pregnancy trimesters, with its concentration increasing from the third trimester to birth. However, low cortisol levels are also quantified during the second trimester, when cortisol is suspected to act mainly in the regulation of the hypothalamus-pituitary-adrenal (HPA) axis [65, 66].

This HPA axis also becomes important during late gestation, during the onset of parturition and the maturation of the fetus for birth. The fetal-placental unit indeed works as a unit, as an endocrine feedback loop is established, dependent on placental human corticotropin releasing hormone (CRH) and fetal adrenal GCs [45]. In this loop, fetal adrenal cortisol increases the production of placental CRH [97–99], which in turn activates CRH receptors and positively feedbacks on cortisol production and triggers DHEA/DHEAS biosynthesis directly or by modulating the fetal adrenal’s response to ACTH [100–103]. Subsequently, estrogen production in the placenta is increased, which ultimately allows for uterine contractions and parturition, and cortisol also supports the maturation of fetal organs and the biosynthesis of prostaglandins [104, 105].

Recent investigations suggest a role of placental CRH in determining term vs. post-term births, showing that CRH R2 receptor levels are lower in post-term placentas [106], and by the association of higher CRH levels in women with a recurrent preterm birth [107]. Other research groups have linked CRH levels to fetal liver blood flow, especially during the stage of late gestation [108], and suggest a link between maternal stress, denoted by depressive symptoms, with placental CRH levels during pregnancy leading to increased neonatal cortisol reactivity [109]. Altogether, the feedback between placental CRH and fetal adrenal GCs is essential to gestation and parturition, and the intricate pathways and cascades that are involved still hold much to be discovered.

Within the context of parturition, the contribution of fetal adrenal cortisol, and placental and fetal tissue 11βHSD enzymes to the maturation of the fetus is also important. 11βHSD2 inactivates cortisol to cortisone while 11βHSD type 1 (11βHSD1) activates cortisol from cortisone. Prior to parturition, cortisol levels increase in the amniotic fluid, due to the increase in placental and, amnion and chorion 11βHSD1 activity (and down regulation of placental 11βHSD2 activity) [110]. Amniotic cortisol is presumably swallowed by the maturing fetus and sustains the maturation of the fetal lungs, both structurally and functionally [111]. Additionally, cortisol also supports the development of the fetal liver and kidney (predominantly functionally) and the gut (both structurally and functionally) [112].

Finally, the adrenal medulla produces catecholamines. While they are not as studied in the context of gestation compared to steroid hormones, there exists an interplay between GC and the medulla [113]. In a study conducted in rats, changes in adrenal medulla function due to administration of dexamethasone (DEX; exogenous GC) during pregnancy showed increased expression of mRNA and protein levels of catecholamine biosynthetic enzymes in the adrenals of both male and female offspring, resulting in increased epinephrine levels in DEX-exposed offspring [114, 115]. It appears that the biosynthetic pathway in the production of catecholamines in the medulla are responsive to exogenous GC stimulation. This is further supported by melanocortin 2 receptor knockout (*Mc2r*^*-/-*^) mice which have adrenal insufficiency and reduced epinephrine levels and expression of catecholamine biosynthetic enzymes [116]. Vice versa, the expression of β2-adrenergic receptors, which bind catecholamines, in all zones of the human adult adrenal cortex, suggests a possible influence of catecholamines on cortical function [117]. It should be noted however, that these findings relate to the adult adrenal and the contribution of the fetal adrenal to these interactions aren’t yet clearly defined.

The proper development of the fetal adrenals and the subsequent proper functioning and steroidogenesis work together in concert to afford the fetus the best chance of survival as a neonate. Unfortunately, abnormal development of the fetal adrenals leading to serious steroid insufficiencies and malformation of organ systems occur and ultimately hinders normal fetal development, as reviewed in the following section.

## Human disorders related to fetal adrenal development and steroidogenesis

Congenital adrenal agenesis is an extremely rare condition, in which the adrenal glands fail to develop [118–120]. Its incidence is unknown and information relating to the pathogenesis is limited [121]. The clinical presentation of adrenal agenesis is also very variable. Affected fetuses may die *in utero* or survive until birth without major problems. The lack of adrenal steroids may however lead to impaired lung and organ maturation late in gestation, and affect the timing of labor [1]. After birth, respiratory distress, pulmonary hypertension, arterial hypotension, hypoglycemia, and electrolyte disturbances are typical signs of an acute adrenal crisis, and, if survived longer, hyperpigmentation and failure to thrive may hint at adrenal insufficiency. However, complete loss of adrenal hormone production of both the cortex and the medulla is not compatible with postnatal life without hormone replacement therapy. Accompanied by adrenal agenesis, additional anomalies are often found in other organs including kidney, lung, spleen, and the vascular system (Table 2). It is also often associated with intrauterine growth restriction.

**Table 2:** Genes involved in adrenal development, selected based on mouse developmental biology studies, and their suggested human correlate according to Online Mendelian Inheritance in Man (OMIM)

Mouse gene name	Phenotype associated with gene inactivation in mouse	Human gene name	OMIM	Human disorder
*Cited2*	Embryonic lethality associated with cardiac malformations, adrenal agenesis, abnormal cranial ganglia, exencephaly, liver dysgenesis, XY sex reversal, eye, lung, and placental defects. Conditional inactivation show failure in maintenance of adult hematopoietic stem cells [17, 18, 157–163].	*CITED2*	614,431 614,433	Atrioventricular septal defects
*Gata4*	Defects of cardiac and pancreatic development, impaired male and female gonadal function [21, 164–167]	*GATA4*	615,542 607,941 614,430 187,500 614,429	Testicular and cardiac anomalies, including atrioventricular septal defects
*Gata6*	Early death at implantation, associated with extraembryonic tissue defects. Conditional inactivation results in adrenal hypoplasia [21, 168]	*GATA6*	614,475 614,474 600,001 217,095 187,500	Cardiac defects included in the Tetralogy of Fallot, pancreatic agenesis
*Nr0b1*	Abnormal reproductive development in the hemizygote, ranging from defects in testes development and spermatogenesis to complete male to female sex reversal, progressive adrenal failure [169, 170]	*NR0B1*	300,018 300,200 300,473	Congenital adrenal hypoplasia with hypogonadotropic hypogonadism
*Nr5a1*	Agenesis of adrenal glands and gonads, defects of the ventromedial hypothalamic nucleus and pituitary gonadotrophs, neonatal lethality [171]	*NR5A1*	612,964 613,957 612,964 612,965 617,480	Haploinsufficiency leads to broad range of 46,XX and 46,XY DSD, ovarian and spermatogenic failure, and rarely adrenocortical insufficiency
*Pbx1*	Late gestational death, hypoplasia or aplasia of multiple organs, impaired hematopoiesis, skin edema, axial and appendicular skeleton defects, absent adrenal glands, impaired development of bone, kidney and pancreas [172–176]	*PBX1*	617,641	Congenital anomalies of kidney and urinary tract syndrome with or without hearing loss, abnormal ears, or developmental delay
*Tcf21*	Lethality around birth due to hypoplastic lungs and kidneys, with abnormal vasculature of these organs and the hemopericardium. Asplenia only in one mouse model [177, 178]	*TCF21*	-	-
*Wnt4*	Perinatal lethality associated with impaired development of the kidney, lung and pituitary gland and female reproductive system. Conditional inactivation results in impaired zG differentiation. [179–184]	*WNT4*	611,812 158,330	SERKAL syndrome, Müllerian aplasia and hyperandrogenism
*Wt1*	Late gestational death, impaired renal, gonadal, adrenal, splenic, pulmonary, cardiac and mesothelial development [16, 19]	*WT1*	194,080 136,680 608,978 156,240 256,370 194,070	Denys-Drash syndrome, Frasier syndrome, Meacham syndrome, mesothelioma, nephrotic syndrome and Wilms tumor

Given that adrenal development is complex, multiple genes may be involved in the pathogenesis of adrenal agenesis [122]. These include transcription factors, signaling molecules, steroidogenic hormones, and extracellular matrix proteins important for adrenal development (see Sect. 2). Although several genes have been revealed by recent developmental studies in, for example knockout (KO) mice models, only very few human disease correlates have been reported so far (Table 2). This may be since many of these genes are also important for the development of other organ systems, which – if not expressed normally during early pregnancy - cause embryonic death.

Because of their common embryonic origin, the adrenal cortex, gonad, and kidney share several molecular developmental pathways, including WT1 and WNT4. However, although reported individuals with *WT1* variants consistently show an abnormal genitourinary development, adrenal agenesis or hypoplasia have not yet been described in these patients, while it is included in the phenotypic spectrum in the KO mouse model (Table 2). Similarly, only one homozygous mutation in the *WNT4* gene has so far been identified in three fetuses of a consanguineous family manifesting with a spectrum of female sex reversal, dysgenesis of kidneys, adrenals, and lungs, therefore named SERKAL syndrome [123]. By contrast, heterozygous *WNT4* variants have been described in at least three young 46,XX women without reported adrenal abnormalities, but a phenotype of absence or hypoplasia of Müllerian duct derivatives (for example, the uterus) and hyperandrogenism is present [124–126].

Adrenal hypoplasia congenita (AHC) and congenital adrenal hyperplasia (CAH) differ significantly from adrenal agenesis as adrenal glands are present but show structural and/or functional aberrations. In AHC and CAH the underlying genetic defects can be identified in up to 80% of cases at present (reviewed in [4, 6, 127–136]). The spectrum of these disorders may be grouped into disorders affecting only the adrenals or having additional effects on sexual development versus syndromic disorders, which show a more complex phenotype with extra associated organ abnormalities. Common to all - they cause primary adrenal insufficiency without major consequences prenatally, but with possible live-threatening adrenal crisis very soon after birth or later in life.

The most common non-syndromic genetic defect causing isolated X-linked AHC is observed with variants in the *NR0B1* gene, encoding the DAX1 transcription factor involved in early adrenal development. Variants in *NR0B1* typically manifest in boys with primary salt-wasting adrenal insufficiency, hypogonadotropic hypogonadism, and infertility. However, if the variant is part of a contiguous gene deletion syndrome on the short arm of the X chromosome (Xp21), patients may also have Duchenne muscular dystrophy and mental retardation [6, 129].

Another rare cause of AHC are variants in *the NR5A1* gene. Although the *Nr5a1* KO mouse shows adrenal and gonadal dysgenesis [24], and the first reported child with a heterozygous *NR5A1* variant had a similar phenotype of adrenogonadal dysplasia [28], follow-up reports showed that variants in *NR5A1* are mostly associated with a very broad range of DSD and reproductive malfunction, and only extremely seldom associated with an adrenal phenotype [30, 129, 137]. In fact, we found less than ten patients with *NR5A1* mutations with a primary adrenal insufficiency with or without DSD in the current literature [28, 29, 132, 138–140].

Several complex syndromes with known and unknown underlying genetic defects are associated with dysgenetic adrenals at birth, but for many of them the pathomechanism of the adrenal phenotype is not fully understood [4, 6, 127–136]. The most recently described ones are the MIRAGE and the IMAGE syndromes. The IMAGE syndrome is usually caused by heterozygous missense variants in the negative cell cycle regulator, cyclin-dependent kinase inhibitor 1 C (CDKN1C). It is characterized by intrauterine growth restriction, metaphyseal dysplasia, adrenal hypoplasia and genitourinary anomalies [141]. The MIRAGE syndrome is either due to gain‐of‐function variants in the growth repressor, sterile alpha motif domain containing 9 (SAMD9) gene [142, 143], or due to biallelic loss‐of‐function variants in the polymerase epsilon‐1 (POLE1, Pol ε) gene [144], which both are important for DNA replication and growth. The MIRAGE syndrome is clinically characterized by myelodysplasia, infections, restricted growth, adrenal hypoplasia, gonadal anomalies and enteropathy.

Furthermore, many inborn errors of metabolism can lead to syndromes with dysgenetic adrenals and/or primary adrenal insufficiency that often do not manifest at birth, but later in life [4, 145]. Perhaps the best known is the X-linked adrenoleukodystrophy, which develops due to mutations in the *ABCD1* gene [146]. However, the most recent discovered disorder in this category is sphingosine-1-phosphate lyase 1 (SGPL1) deficiency, in which recessive loss-of-function *SGPL1* mutations cause syndromic adrenal insufficiency associated with steroid-resistant nephrotic syndrome, variably accompanied by ichthyosis, primary hypothyroidism, cryptorchidism, immunodeficiency and neurological anomalies [147–149]. The SGPL1 enzyme catalyzes the final breakdown of sphingolipid S1P, which regulates cell migration, differentiation, and survival, together with angiogenesis and development. The pathogenesis of SGPL1 deficiency includes both compromised adrenal development and disrupted steroidogenesis, however it is currently being studied in more detail [150].

The group of CAH comprises genetic defects of adrenal cortisol biosynthesis [4, 6, 127–136]. These do not *a priori* affect adrenal development, but may impact secondary organ structures as seen with steroidogenic acute regulatory protein (StAR) related lipoid CAH [151]. As genes involved in early steps of steroid biosynthesis, such as *StAR, cytochrome P450 side-chain cleavage (CYP11A1), HSD3B2, CYP17A1, POR, cytochrome b5 (CYB5)* are common to both adrenal and gonadal steroidogenesis, they may manifest at birth with primary adrenal insufficiency and 46,XY undervirilization due to steroid hormone deficiencies of prenatal onset. By contrast, genetic variants in *CYP21A2* and *CYP11B1* cause cortisol deficiency, accompanied by adrenal androgen excess [4] - affected 46,XX fetuses have CAH and show genital virilization at birth due to intrauterine exposure to high androgen levels that the fetal-placental unit could not metabolize (Fig. 2). Similarly, variants in genes directly involved in fetal-placental steroidogenesis, for example *POR* and *CYP19A1*, may lead to atypical sexual developments in chromosomal male and female fetuses with or without disturbed adrenal postnatal function, and may even virilize mothers during pregnancies [4].

## Ongoing research in the field, unsolved questions and our perspective

Knowledge on the biology of fetal adrenal development and function has markedly advanced in recent years through the application of novel models and approaches and newer technical possibilities. Most notably, the greater details entailing the spatio-temporal network of genes which collaborate in the early development of the human fetal adrenals during the first trimester has been elucidated (Fig. 1), while adrenal development throughout the subsequent gestational trimesters remain more obscure - especially the switch(es) which result(s) in the transition from the fetal adrenal organ to the postnatal adrenal after birth. Likewise, human fetal adrenal steroidogenesis throughout gestation is perhaps best studied in the first trimester and at birth for obvious ethical reasons concerning biomaterial availability. Regardless, recent advances in steroid profiling have questioned the textbook knowledge and revolutionized our understanding of steroid biochemistry regarding androgen biosynthesis and metabolism [71, 72, 77]. This resulted in the re-evaluation of the steroid biosynthesis capacity of the fetal-placental unit (Fig. 2), which is still ongoing.

In line with a concerted effort among researchers to better understand adrenal development, a recent work commented on earlier in this review [8] illustrated how tackling the mechanisms of early adrenal development in humans using established and state-of-the-art techniques, including immunostaining and single-cell transcriptomics, can support the investigation of key cellular and molecular dynamics with an unprecedented resolution. A crucial aspect highlighted by Cheng et al. are main differences in the adrenal developmental mechanisms between human and mouse, the laboratory species in which adrenal development and physiology has mostly been studied. While this poses serious caveats about the use of rodents as models of human adrenal physiology, it can also shed light on the reasons why inactivation of ortholog genes results in different phenotypes between the two species. The *NR5A1* and *WT1* genes are arguably the most paradigmatic, in that their inactivation in humans mostly results in genitourinary abnormalities, with rare or no impact on the adrenals, while in mice it affects adrenal development with high penetrance [17, 26, 27]. Altogether, the work by Cheng et al. suggests that a deeper understanding of adrenal fetal maturation in humans may greatly benefit from a granular analysis at serial developmental stages [8]. A clear example of this kind of analysis is given by the work of the Achermann’s group [152], who dissected the unique genetic components in the developing adrenal compared to other steroidogenic and non-steroidogenic tissues. Their transcriptomic profiles revealed adrenal-specific genes throughout multiple developmental stages between 6 and 10 wpc, thus paving the road for the identification of new culprits for adrenal a-/dysgenesis and contributes (potentially) to explaining the remaining 20% of orphan AHC and CAH cases. On top of this, advancements in the generation of patient-derived differentiated adrenal cells through the intermediate stage of iPSCs, or directly originated from cells of mesenchymal origin, hold promise for further insights on adrenal malformations. However, protocols for zonal-specific cell differentiation have still to be refined, and the studies for the regeneration of adrenal organoids, which would dramatically boost adrenal investigation, are still ongoing worldwide [33, 153].

Our review of recent literature also highlighted an increased effort to place adrenal development and physiology within the larger context of hormonal regulation. For instance, mouse studies revealed that bone-derived osteocalcin modulates fetal adrenal homeostasis, leading to the definition of a bone-adrenal endocrine circuit [154, 155]. Osteocalcin injections increased circulatory corticosterone (to a similar extent as observed with ACTH) and aldosterone (also observed in rhesus monkeys), together with the upregulation of *Cyp11b1* and *Cyp11b2* expression. Osteocalcin-deficient (*Ocn*^*-/-*^) mice born from *Ocn*^*-/-*^ parents showed lower corticosterone and aldosterone levels and decreased expression of *Cyp11b2* and *Cyp11b1* compared to WT mice and *Ocn*^*-/-*^ mice born from a cross between *Ocn*^*+/-*^ parents, suggesting that osteocalcin in embryos may regulate adrenal steroidogenesis and adult adrenal function. These findings establish the bone-adrenal endocrine circuit as an important research topic for future investigations. Of note, the influence of thyroid hormones on fetal adrenal development are also an avenue of future research inquiry, with findings revealing that congenital hyperthyroidism or hypothyroidism modify the development of the cortex and its steroidogenic activity, while also altering the medullary gene expression profile [156]. Finally, recent literature makes way for the investigation into the origin and potential role of the C11-oxy androgens in the fetal-placental unit - especially when considering their levels in the (term) fetal-placental unit [71, 77], and that their levels during the critical period of sex differentiation are still not known. Permitted that additional critical time points during gestation can be investigated, steroid profiling of these androgen precursors and androgens at these critical time points might shed light on their function in fetal development.

## Abbreviations

DHEA-S: dehydroepiandrosterone-sulfate
3βHSD1: 3β-hydroxysteroid dehydrogenase type 1
17βHSD: 17β-hydroxysteroid dehydrogenases
CYP19A1: cytochrome P450 aromatase
DHEA: dehydroepiandrosterone
A4: androstenedione
T: testosterone
E2: estradiol
CYP3A7: cytochrome P450 family 3 subfamily A member 7
E3: estriol
E1: estrone
GC: glucocorticoids
PROG: progesterone
CYP11A1: cytochrome P450 cholesterol side-chain cleavage
CYP17A1: cytochrome P450 17α-hydroxylase/17,20-lyase
CYP21A2: cytochrome P450 21-hydroxylase
CYP11B1: cytochrome P450 11β-hydroxylase
ACTH: adrenocorticotropic hormone
SRD5A2: steroid-5α-reductase type 2
DHT: dihydrotestosterone
AR: androgen receptor
CYP11B2: cytochrome P450 aldosterone synthase
11βHSD: 11β-hydroxysteroid dehydrogenase
11OHA4: 11β-hydroxyandrostenedione
11KA4: 11keto-androstenedione
11KT: 11keto-testosterone
POR: cytochrome P450 oxidoreductase
PORD: cytochrome P450 oxidoreductase deficiency
PREG: pregnenolone
17OHPREG: 17α-hydroxypregnenolone
StAR: steroidogenic acute regulatory protein
SULT2A1: sulfotransferase
STS: sulfatase
DSD: disorders/differences of sex differentiation
17OHPROG: 17α-hydroxyprogesterone
DEX: dexamethasone
iPSCs: induced pluripotent stem cells
Ocn: osteocalcin
MC: mineralocorticoids
DZ: definitive zone
FZ: fetal zone
TZ: transition zone
wpc: weeks-post-conception
dpc: days-post-conception
zF: zona fasciculata
zR: zona reticularis
zG: zona glomerulosa
AGP: adrenogonadal primordium
GATA4: GATA binding protein 4
WT1: Wilms’ tumor 1
CITED2: Cbp/p300-interacting transactivator 2
GP: gonadal primordia
AP: adrenal primordia
Tcf21: transcription factor 21
Gli1: glioma-associated oncogene 1
NR5A1: nuclear receptor subfamily 5 group A member 1
SF1: steroidogenic factor-1
HOX: homeobox
FAdE: fetal adrenal enhancer
WNT: Wingless/Integrated
KO: knockout
AHC: adrenal hypoplasia congenita
CAH: congenital adrenal hyperplasia
CDKN1C: cyclin-dependent kinase inhibitor 1 C
SAMD9: sterile alpha motif domain containing 9
POLE1: polymerase epsilon‐1
SGPL1: sphingosine-1-phosphate lyase 1
CYB5: cytochrome b5
CRH: corticotropin releasing hormone
HPA: hypothalamus-pituitary-adrenal
RoDH: retinol-like dehydrogenase.
